# Quality Improvement Initiative to Improve Uptake of an Alternative Call Management Strategy for Home-Based Hospice Care

**DOI:** 10.1089/pmr.2024.0107

**Published:** 2025-05-19

**Authors:** Winnie Choo, Zhi Zheng Yeo, Xiang Yi Chen, Lily Li, Lan Miao, Xiao Juan Lyu, Poh Heng Chong

**Affiliations:** HCA Hospice, Singapore, Singapore.

**Keywords:** call diversion, collaboration, communication, home hospice, quality improvement, telephone

## Abstract

**Background:**

In home-based hospice care, frontline nurses frequently need to take unscheduled incoming calls while out in the field. This interrupts critical tasks and disrupts patient rapport, potentially lowering care quality for patients. At HCA Hospice in Singapore, the 30 frontline nurses could receive up to 135 calls/day. In mitigation, a telemedicine call-center system (MediHELP) was conceived in September 2023 for frontline nurses to divert incoming calls to a dedicated team for timely management remotely. However, call diversion to MediHELP remained low. A Quality Improvement project (December 2023–July 2024) was designed to catalyze the process change.

**Methods:**

Using the plan-do-study-act (PDSA) model, we aimed to increase call diversion rates to MediHELP by 50%. Initial root cause analysis, corroborated with surveys and focus groups, revealed key challenges: inconsistent processes, inconvenient diversion procedures, and lack of awareness. The first PDSA cycle focused on developing a standardized communication protocol with nurse input, while the second cycle broadened operational hours and improved outreach efforts. Outcomes were evaluated by examining call diversion rates and conducting feedback surveys among stakeholders to assess confidence in the MediHELP team and perception of its effectiveness.

**Results:**

Implementations led to a significant increase in call diversions (from 11% to 65%), achieving 600 calls per month within six months, passing the targeted diversion rate of 50%. Home care nurses reported increased confidence with the MediHELP team, improvement in its perceived effectiveness, and acknowledged that call diversion had led to less stress and greater focus at work.

**Conclusion:**

Successful implementation of a new initiative that reduced nurse burden was achieved by addressing workflow barriers. This initiative could support the future expansion of home care capacity. Additionally, MediHELP services would be extended to patients under day-hospice support within the organization.

## Background

In the home care setting, care delivery is usually organized on a primary nurse-led model, where a home care nurse is the main point of contact for the patient and their caregivers.^1,2^ As the main point of health care contact, home care nurses typically provide their direct phone number to their patients for calling and texting. In this model, nurses remain contactable at all times during work hours; many would not silence their phones, even when in the middle of home visits or important tasks.

Incoming calls can come from patients, their family caregivers, or other health care professionals for advice, updates, and urgent action, running the gamut from prescription requests to new distressing symptoms. However, studies in other settings suggest that calls and texts adversely impact worker efficiency and patient safety, by interrupting tasks that require focus.^3,4^ In home-based care, unscheduled incoming calls can disrupt important conversations and nursing procedures during the home visit. If the issue from the incoming call was urgent, the home care staff might also feel compelled to make an unscheduled visit, further disrupting their planned visits for the day. These interruptions may be deleterious to all stakeholders. frontline staff reported that rapport with patients and family was affected, and the cognitive load was high.^5^ On the contrary, patients and families who are calling for help experience slower response times from their primary nurse. To address the drawbacks of frontline nurses fielding calls while out making home visits, alternative methods of managing incoming calls should be explored.

A telemedicine-based call-center model may be a viable solution. In this model, incoming phone calls to home care nurses while they are in the field (frontline nurses) would be diverted to a team of nurses who would work remotely to respond to these calls. This approach would not only reduce their strain but it may also improve the response time to patients and families making the calls, thereby improving patient outcomes, such as time from symptom onset to medical contact.^6,7^

However, such a model for home care had not been explored in existing literature. To date, there has not been any study on the implementation of a telemedicine center model to manage incoming calls from patients in home-based palliative care. Moreover, there was no information on how existing services could transition from the conventional “call primary nurse” model to this alternative “call-center model.”

### Setting and clinical context

In Singapore, the majority of hospice and palliative care needs at home are supported by HCA Hospice (HCA). HCA is a nonprofit charitable health care service helmed by palliative care specialists, including nurses, physicians, medical social workers, and allied health professionals (e.g., art therapists and spiritual care counselors). While the service is primarily focused on home-based end-of-life care, HCA also maintains two day-hospice centers as a source of respite care. To resolve any palliative care issues that may arise at home, all HCA patients and caregivers were given the phone number of their primary nurse (the frontline nurse) to call during the day, and an emergency helpline number to call after office hours to reach an on-call physician for help.

Palliative home care nurses in our context are distributed in five satellite offices based on geographical location. They work office hours (8:30 am−5:30 pm) with a typical schedule being the following: (1) begin the day with a multidisciplinary meeting to discuss and plan for complex or urgent patient cases; (2) contact patients or caregivers to schedule (four to five) home visits for the day; (3) during home visits, take patient history, conduct physical examinations, discuss management plans with patient or caregivers, update family members, and perform procedures such as dressing changes or changes of feeding tubes; and (4) return to office at the end of day to record case-notes into the electronic medical records (EMRs).

Every year, the organization admits up to 3500 new patients who have a prognosis of one year or less. Patient load is pegged against a patient-to-nurse ratio of 35:1. As Singapore is poised to become a “super-aged society,”^8^ the number of patients HCA is expected to serve will likely increase as the government encourages for more patients to access health care within the community. Under the previous “call primary nurse” model, frontline staff faced frequent unscheduled incoming calls, with approximately 135 calls per day among 30 home care nurses during their working hours.

### Development of the MediHELP “call-center model”

An initiative to establish a “call-center model” started in September 2023. The call center (named “MediHELP”) consisted of a team of three full-time equivalents of senior palliative care nurses and one physician. This pioneer team developed the first set of protocols for managing incoming calls, including (1) the preparation of scripts to explain why patients were then being diverted, (2) procedures to advise symptom management over telephone, and (3) processes to update case-notes after the teleconsultation.

Unlike frontline nurses, MediHELP nurses were not assigned to be the primary nurse of any patient; their role was to be stationed in headquarters to receive incoming phone calls to the service and to resolve immediate issues raised during these calls. Phone calls would be diverted from frontline nurses during the time they were occupied with making home visits, between 10:00 am and 4:00 pm. To enable call diversion, the frontline staff were to enable “call forwarding” in their phones to divert incoming calls to MediHELP; this setting still allowed the frontline staff to make outgoing calls.

When receiving these calls, the MediHELP team would provide teleconsultations to resolve the callers’ issues. If urgent and requiring immediate attention, they would either conduct a video consultation to allow for visual assessment or make *ad hoc* home visits. For nonurgent issues, the team will provide advice over the phone to the caller. These patient interactions by the MediHELP team are documented in the EMRs; frontline teams would then access these records at the end of the day to get updates about their cases and manage accordingly.

Before the clinical or operational impact of the MediHELP service could be meaningfully evaluated, it was critical to first establish whether this call-center model could be successfully adopted as an alternative to the conventional “call primary nurse” workflow. At the project’s outset, the MediHELP service was underutilized; three months after launch, MediHELP was only receiving an average of 15 calls per workday—approximately 11% of the expected 135 calls on a workday. This would render it statistically and practically unfeasible to assess downstream effects on patient outcomes, staff workload, or efficiency.^9^

Thus, this quality improvement (QI) project prioritized addressing barriers to call diversion as a prerequisite for future impact evaluation. By systematically improving the uptake of MediHELP, we aimed to create a stable foundation for subsequent evaluation. A target call diversion rate of 50% was set, as it would be a 4.5-fold increase from the current 11% adoption rate, demonstrating meaningful workflow integration without being overambitious. This goal would also allow for measurable operational changes in the future (e.g., reduced frontline nurse interruptions).

## Materials and Methods

For this project, a QI approach was employed as it allows for a systematic process for identifying the underlying cause and developing solutions.^10^ The goal was to understand why frontline nurses were not diverting their calls to MediHELP, and to test if solutions did subsequently improve call diversion rates. A secondary objective was to assess staff perceptions of the transition. This project was reported with reference to the Revised Standards for Quality Improvement Reporting Excellence checklist (SQUIRE 2.0).^11^

### Team composition and design

The QI project group included the original members of the MediHELP team (three registered nurses and one doctor) who acted as the core team. We were supported by one medical-administrative and one research staff, HCA’s Medical Director, and the Assistant Director of Nursing. The core team collected data, organized meetings, and iterated on the service’s model. Other members contributed resources and input on the interventions’ design and implementation, such as purchasing and coordinating the installation of a Private Branch eXchange system to handle multiple inbound and outbound lines and call routing.

### Root cause analysis

A root cause analysis (RCA) was conducted by our QI team to systematically identify factors contributing to low call diversion rates.^12^ Between December 2023 and January 2024, reasons for the poor diversion rate were solicited *via* an anonymous self-administered online survey, one-on-one meetings, and focus groups with frontline nurses and their team managers. Twenty-five nurses out of 30 nurses responded to the anonymous online self-administered survey. Focus groups were conducted with five different teams, consisting of an average of six nurses per team. The RCA was conducted by the QI team. Data for the RCA were collated and thematically grouped into categories, which were organized in a fishbone diagram.

### Plan-do-study-act

Based on the RCA, the team then utilized the plan-do-study-act (PDSA) methodology to systematically implement and assess various interventions aimed at improving the uptake of the MediHELP.^13^ PDSA cycles provide a structured framework for planning interventions (plan), executing these planned actions (do), observing and analyzing the results (study), and refining the strategy based on observed outcomes (act).

### Evaluation and measures

To assess the effectiveness of the interventions, a mixed methods evaluation strategy was employed, using both qualitative and quantitative measures to assess intervention impact.^14^ The primary outcome measured was the number of incoming phone calls received by the MediHELP team during office hours. This outcome was chosen as a direct measure of frontline staff successfully transitioning to the MediHELP model; a monthly aggregate measure was used as it would be more reliable than daily numbers, which tended to fluctuate.

To this end, the MediHELP team separately recorded every incoming phone call they received to determine the “actual” number of incoming calls to MediHELP. However, one limitation was that this total could not be compared directly with the number of phone calls as documented in the EMR by frontline staff. This is because frontline nurses typically do not record every incoming phone call from the same patient on the day as separate case-notes; they would instead group these calls into one case-note, documenting from the first incoming call to the last call that resolved the primary issue. The MediHELP nurses also documented in a similar fashion in the EMR. Hence, call diversion rates were calculated by tallying the proportion of “phone-call case-notes” in the EMR that were logged by MediHELP.

The secondary measure was the frontline nurses’ perception of the MediHELP team’s effectiveness. Two rounds of feedback surveys were conducted: (1) one presurvey at the start of PDSA cycle 1 to capture baseline data, and (2) a postsurvey conducted near the end of the second PDSA cycle (May−June 2024). The surveys were online, anonymous, self-administered, conducted over Microsoft Forms. Respondents were asked to (1) report the typical daily number of incoming calls they were receiving before and after MediHELP implementation, and then (2) rate two statements on a Likert scale of 1 − 5: “How concerned are you about forwarding your calls to MediHELP?” and “Is MediHELP effective in addressing your patients’ calls?”. Free remarks were also recorded for qualitative analysis to extract common ideas in their responses.^15^

### Analysis

Quantitative and qualitative analyses were conducted to evaluate the impact of interventions. For the primary outcome, monthly call case-notes logged by the MediHELP team were analyzed using descriptive statistics and trends to assess progress toward the 50% diversion target. Secondary outcomes were analyzed by comparing pre- and postsurvey responses: (1) differences in staff-reported number of calls pre- and postimplementation were tested with Student’s *t* test; (2) Likert scale ratings were summarized using Fisher’s exact test to evaluate shifts in perceptions. Free-text remarks were thematically analyzed: responses were coded inductively by the team to identify recurring themes, with discrepancies resolved through consensus. Mixed methods integration was employed to contextualize quantitative trends with qualitative feedback.^14^

### Ethics declaration

This initiative did not undergo formal review by an institutional review board, as it focused on internal process improvement without patient-specific research and thus is exempt.^16^ No patient data was collected, and feedback from the ground staff was collected without any personal identifiers. No special funding was received.

## Results

### Root cause analysis

**FIG. 1.**
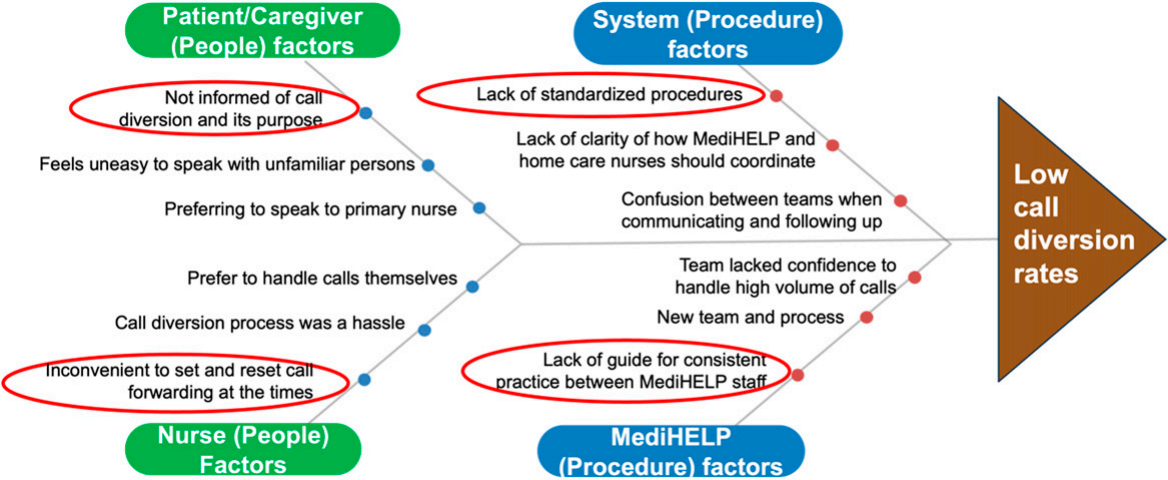
Fishbone diagram of factors for low call diversion rates to MediHELP service.

Figure 1 shows the final fishbone diagram for the RCA. Four main categories were identified: systems (procedure) factors, MediHELP (procedure) factors, patient/caregiver (people) factors, and nurse (people) factors:

(1) **System (procedure) factors:** Feedback revealed differences in expectations between the MediHELP team and the frontline nurses, particularly with regard to communication and management. Moreover, the MediHELP team faced difficulties planning actions when reviewing nursing documentation that had insufficient information. There was a lack of defined roles, which could lead frontline nurses to sometimes expect greater assistance or sometimes feel excluded from decision making. This led to frustration and reduced trust on both sides, leading to frontline nurses not wanting to divert calls to MediHELP.
(2) **MediHELP (procedure) factors:** There was a lack of guidance for the MediHELP staff to standardize practice. While the MediHELP team consisted of experienced nurses who were previously frontline nurses, they were new to their dedicated role in managing incoming calls. Feedback surfaced inconsistencies in MediHELP team’s practice. For example, while some MediHELP members used urgent calls to update frontline nurses, others relied on text messages or expected nurses to check their documentation at the end of the workday. This reduced confidence in the MediHELP service, reducing willingness to divert calls.
(3) **Nurse (people) factors:** Although the home care services’ office hours were between 8:30 am and 5:30 pm, MediHELP coverage was originally only active between 10:00 am and 4:00 pm. Frontline nurses needed to manually turn on “call forwarding” function in their phone settings for call diversions. This caused nurse workflow challenges, as nurses often found it a hassle to set call forwarding on a daily basis.
(4) **Patient/caregiver (people) factors:** There was an awareness gap where patients and caregivers were not routinely informed that incoming calls would be diverted for the reason of avoiding interruptions during their daily work. As they were not prepared to speak to MediHELP, they would refuse to speak with the team and asked to contact their frontline nurses directly. When this happened, caregivers became accustomed to contacting their primary nurses *via* ways that bypass call diversion (e.g., using WhatsApp calls or text messages).

The prioritization of factors for intervention was guided by both the frequency and impact of the identified issues from the frontline nurses’ feedback. Three key priorities were identified:

(1) There was a need to standardize communication and management protocols to ensure smooth collaboration,
(2) MediHELP’s operational hours needed to be expanded to address the hassle of daily diversions, and
(3) A standardized outreach program was needed to raise patient and caregiver awareness regarding the new call diversion protocols and their benefits to encourage adoption of MediHELP.

### Iterative implementation of MediHELP

The iterative refinement of MediHELP was guided by two PDSA cycles, each addressing distinct barriers to call diversion. Detailed phase-specific actions and outcomes are summarized in Table 1.

**Table 1.** Plan-Do-Study-Act Cycles to Increase Call Diversion to MediHELP

PDSA cycle	Timeline	Plan (focus)	Do (key actions)	Study (outcomes)	Act (lessons learned)
Cycle 1	December 2023− February 2024	Align communication and protocols	Codesigned communication protocol with frontline Registered Nurse (RN) input.	No significant increase in call diversion rates.	Trust-building requires time.
			Established documentation standards.	20% improvement in RN confidence in MediHELP.	Operational barriers (e.g., call forwarding) hindered adoption.
			Introduced chat groups for updates.		
Cycle 2	March 2024−May 2024	Improve ease of use and uptake	Extended operational hours (8:30 a.m. to 5:30 p.m.).	Call diversion rates exceeded 50% by May 2024.	Convenience (e.g., default call forwarding) and awareness drive adoption.
			Added staff to handle increased demand.	35% reduction in RN interruptions.	
			Educated patients/caregivers during admission.		

Cycle 1 was conducted between December 2023 and February 2024. The objective was to address fragmented workflows and inconsistent documentation. The team designed a standardized protocol for updating the staff *via* documentation and nonintrusive chat notifications. The efforts helped to build the staff’s trust in the MediHELP service. However, diversion rates remained low due to other operational barriers.

Cycles 2 was conducted between March 2024 and May 2024. This cycle focused on the cumbersome process of call forwarding at inconvenient times, as well as the reluctance of family members who were unfamiliar with MediHELP staff. The call diversion process was revised by expanding MediHELP hours, which helped to reduce manual effort, while a new step to introduce MediHELP was added to the patient orientation package. With these changes, diversion rates began to increase.

### Impact

The implementation of the QI initiatives yielded significant improvements in call diversion rates and the everyday experiences of home care nurses, as summarized below:

#### Experience of home care nurses

In the first survey, home care nurses had reported that they typically received an average of 4.8 incoming calls per day prior to MediHELP’s launch. By June 2024, calls subsequently reduced to 1.9 calls a day (a 2.67 times reduction; *p* < 0.001). There was also an improvement in nurses’ perception of MediHELP’s effectiveness and their confidence to divert calls (Table 2); by the second PDSA cycle, the majority of nurses (>80%) rated MediHELP positively for both items.

**Table 2.** Reported Outcomes From Frontline Nurses Who Diverted Calls to MediHELP

	Baseline (*n* = 25)	PDSA cycle 2 (*n* = 27)
Confidence in MediHELP team*		
Not concerned about diverting calls to MediHELP (4 or 5)	20 (80.0%)	26 (96.3%)
Concerned about diverting calls to MediHELP (3 or below)	5 (20.0%)	1 (3.7%)
Rating of MediHELP’s effectiveness*		
Rated MediHELP effective or very effective (4 or 5)	12 (48.0%)	22 (81.5%)
Rated MediHELP somewhat effective or not effective (3 or below)	13 (52.0%)	5 (18.5%)

^*^ Fisher’s exact test found a significant difference between baseline and PDSA cycle 2 for both confidence in MediHELP team and rating of its effectiveness.

PDSA, plan-do-study-act.

Analysis of the free-text comments showed that frontline nurses found MediHELP had reduced their sense of stress during work hours and had helped to reduce disruption during their home visits (Box 1). Moreover, when nurses were on leave or off-duty, they felt confident that their patients could call MediHELP, rather than imposing on other colleagues to help manage calls. Distressing symptoms were managed in a timelier manner, as the MediHELP team was available to address these symptoms. As MediHELP could also conduct *ad hoc* home visits to see patients for sudden issues, frontline nurses also shared that they were able to avoid canceling preplanned visits.

Box 1. **Excerpts of frontline nurses’ feedback on the impact of MediHELP**

“Very helpful to me; and for the patients to have that ease of mind that there is always someone from HCA to answer their call for help.”

“Reduce disruption during home visits…also has helped to reduce some of our heavy workload.”

“Able to assist in crisis: the patient was alone at home during a symptom crisis; family was overseas, and I was unable to rush down. But MediHELP was able to contact the patient to support with video and voice calls.”

“When [MediHELP] returned calls, they refer to my notes and help to address/assist accordingly.”

“Very good when I’m [on leave]; I will divert MediHELP so I don’t burden my team with the phone calls.”

#### Call diversion rates

**FIG. 2.**
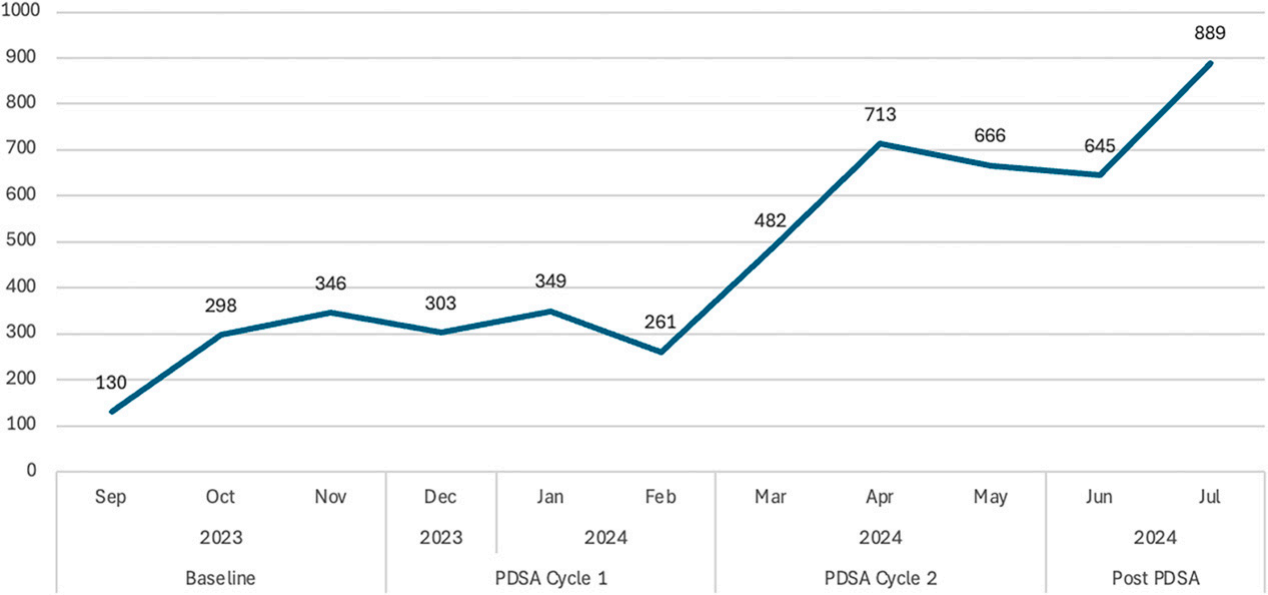
Trend of actual number of calls answered by MediHELP.

Figure 2 shows the actual number of calls MediHELP answered between September 2023 and July 2024. At baseline (September−December 2024), the MediHELP team answered an average of 258 calls per month. During the first PDSA cycle, number of calls marginally increased to 304 calls per month. However, after the second PDSA cycle was conducted, the number increased to an average of 620 calls within the first three months. This increasing trend persisted in July 2024, during which the MediHELP team averaged 30 calls per workday, double the initial number of calls diverted to MediHELP during baseline.

**FIG. 3.**
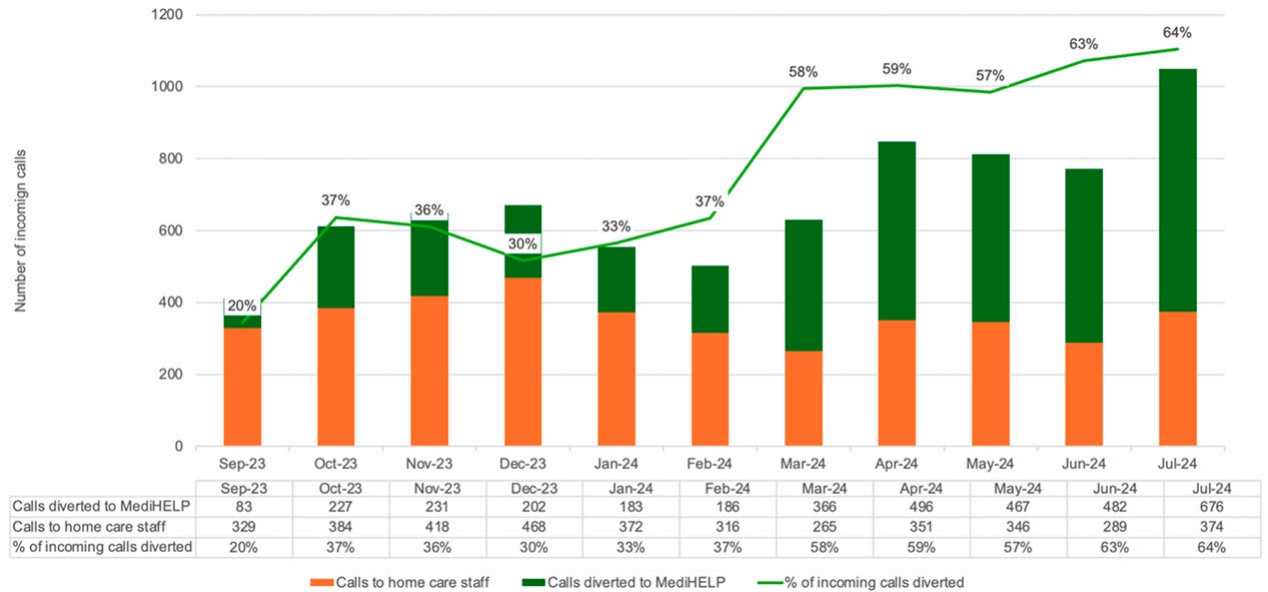
Trend of call diversion rates as counted using phone-call case-notes in the electronic medical records.

Figure 3 shows the total number of documented case-notes of incoming patient-related phone calls during the same period, grouped by those by MediHELP and those by frontline staff. Results are aligned with Figure 1. By March 2024, the proportion of incoming calls diverted to MediHELP reached the target of 50% and achieved 65% from July 2024. This was a 3.25-fold increase from baseline.

## Discussion

Findings showed that the QI efforts did improve the rate of call diversions from frontline nurses to MediHELP, which indicates a greater uptake and acceptance of the alternative call management system. Feedback from staff have found that call diversions do contribute to stress reduction and improved focus on patient care.^17^ However, to ensure successful implementation, several factors must first be fulfilled.

First, this initiative reinforced the importance of structured follow-up mechanisms to ensure continuity of care, early implementation revealed gaps in inter-team communication, particularly during the transition from the previous model to this new one. Effective coordination and inter-team performance require growing mutual trust between all players. This is especially true in roles with high virtuality (where members are not able to collaborate face-to-face), and with high task interdependence (where team members rely on each other).^18^ Although the nurses in MediHELP were experienced in being frontline nurses in the past, their new role in prioritizing new exigencies required additional training to allow them to function well. The refinement and standardization of protocols and expectations may have contributed to the improved confidence of the frontline nurses to divert their calls to the MediHELP team, which was reflected in the change in responses in the survey.

Another area of learning was the importance of reducing friction to transition. During the first PDSA cycle, while approval of MediHELP did increase, call diversion rates did not improve significantly. One major explanation for this lack of significant increase was the initial hassle involved in being part of the call diversion system. When MediHELP was only operational between 10:00 am and 4:00 pm, any nurse who wanted to utilize the system had to manually set call forwarding on a daily basis. Moreover, they had to do so during their home visit timings, when they would have started being occupied. This made the new process cumbersome and difficult to onboard, which led to the staff becoming reluctant to change from their established habit of taking calls from patients while making home visits.

The key to addressing this was to increase the convenience of MediHELP’s call diversion. Increasing convenience and reducing complexity had been identified to be an important factor for improving uptake of a new process.^19^ In the second PDSA cycle, the MediHELP team addressed this issue by increasing their manpower and allowing call diversions to start from 8:30 am to 5:30 pm. By doing so, frontline nurses could now effectively forward their incoming calls to MediHELP by default; only disabling call forwarding when they wish to receive incoming calls directly. This improvement made call diversion much less disruptive and more convenient. Additionally, the groundwork laid during the first cycle likely contributed by gradually building staff confidence in the reliability and utility of the MediHELP system. As trust in the process grew, nurses became more comfortable with leveraging MediHELP for call management.

There was also a need to keep patients and their caregivers up-to-date of this new process. When primary nurses routinely informed caregivers of the routine diversion of calls to MediHELP team, families were less surprised or unprepared to speak to MediHELP colleagues, which would further contribute to the higher number of incoming calls to MediHELP.

The limitations of this QI initiative are multifaceted and warrant careful consideration. First, while part of the process involved notifying caregivers about call diversions, there was no systematic data collection on whether caregivers found this process beneficial or supportive. Future efforts should involve examining the perspectives of patients and their families to better understand their reception and overall satisfaction with the call diversion process, which could further enhance service quality. Second, the findings and interventions may have limited generalizability, as they were focused specifically in improving the rate of call diversions, and not on the effectiveness of the model itself on improving patient or staff experience (although limited evidence of staff experience was collected in this QI project). Variability in practices, resources, and team compositions in other settings could impact the replicability of the results.

## Conclusions

This initiative demonstrates the importance of mutual trust and improving convenience to encourage the uptake of a new process. Although the initial implementation of projects may have a disappointing start, this QI initiative demonstrated that, with targeted changes, positive improvements can be achieved to enhance the service rendered to all stakeholders in the palliative care system.

## Abbreviations Used

EMR: electronic medical records
HCA: HCA Hospice
PDSA: plan-do-study-act
QI: quality improvement
RCA: root cause analysis
RN: registered nurse
